# Round the Hospitals

**Published:** 1916-02-19

**Authors:** 


### Round the Hospitals.

We have had two letters this week from matrons of important children's hospitals, one of which will be found in the Editor's Letter-Box. We invite correspondence on the point raised, which is the special value of the faithful service rendered by sisters and fully trained nurses who remained at their posts in these institutions and did not desert them for work for the war. The truth is that so-called war work has become a craze with many people who, we fear, may thereby waste their energies in fields for which they are not well fitted, to their own loss and that of the country. At this time every man's and woman's duty is to do the next thing. The best of the race do not thirst after decorations or medals, but desire in these strenuous times to do their duty faithfully by rendering loyal, if humble, service, content to know "some there be which have no memorial."

Miss Emily Barry, matron of the Huddersfield Royal Infirmary, has sent us a most interesting letter, in which she expresses great pleasure at having The Hospital's assurance that all the cheerful, ready spirit of help exhibited by the staff in so many voluntary hospitals, which have wounded soldiers under treatment, will receive, there is good reason to hope, some recognition in due course. Five hundred wounded men direct from the Front have been nursed at the Royal Infirmary, which is now affiliated with the Huddersfield War Hospital recently opened. The Royal Infirmary will continue to offer facilities for the instruction of V.A.D. members, and a great deal of massage work is being done there for the wounded. One of Miss Barry's sisters, Annie Charlesworth, is sister to a clearing station in France, and Nurse Eleanor Sturdy is also working there with other nurses from this institution. We shall be glad to have similar items of news from every hospital.

Cardiff, through King Edward VII.'s Hospital, is one of the most active voluntary hospital centres for war work. Its matron, Miss E. A. M. Wilson, R.R.C., apart from her work as matron of this hospital, is principal matron of the 3rd Western General, which is divided into many sections, and requires a good deal of her time and attention. Seven sections of this hospital are situated in practically seven hospitals, as each is some distance apart from the other, and in addition there are three schools and houses occupied as nurses' quarters. An eighth section of the 3rd Western Hospital is situated at Newport, which contains over 700 beds, and a ninth section is at Neath, with 330 beds. These facts will show what a very busy life Miss Wilson must lead, for she is responsible for the nursing staff for all these numerous hospitals, as well as for that of King Edward VII.'s. Altogether the 3rd Western Hospital consists of over 2,000 beds, which seem to have a continuous tendency to grow and increase. Anyone who realises the immense value of the splendid work here recorded will readily agree that those who are working in voluntary hospitals, and putting forth their utmost energy in the nursing and care of wounded warriors, have a great claim upon the gratitude of the nation and the recognition of those who have it in their power to mark their sense of the high value placed upon such service.

A senior and most interesting provincial matron, —an old St. Thomas's sister—Miss A. M. Messum, has done a splendid work at the Canterbury Hospital. One good effect of the reception of wounded men at Canterbury has been the recognition by the governors of the paramount necessity of providing a lift at a cost of £600, which was quickly subscribed. This hospital has further gained a fine balcony, where some officers are under treatment. A further addition has been the *x*-ray installation, and these three recent improvements, due to the war, as the report testifies, have combined to add immensely to the efficiency of the working of the Canterbury Hospital.

We are glad to be able to record the successful treatment and happy fate of those of our soldiers who have suffered the loss of a limb or limbs since the war began. The matron of the Huddersfield Royal Infirmary writes: "We had evidence the other day of the excellent and useful work going on at Roehampton for the soldiers. A man, whose leg had to be amputated in spite of every effort on the part of the surgeon to save it, came to see us, walking wonderfully, and able to get in and out of tramcars, etc. He looked so well and so happy, and displayed the wonders of his artificial leg with great pride, and told us how he was expecting to return to his work at the Post Office within a week or two. With him came his wife, who a few months ago came to see him as his *fiancée*, and then went away weeping at the poor prospect of ever being his wife. Now she is a happy and proud woman."

Many of our readers must have been shocked at the cases which have been published in the papers in which soldiers who have been employed in the trenches in Flanders for many months, and have in consequence returned home permanently invalided through illness acquired in the discharge of their duty, due to the exposure and trying conditions under which they had to serve, have been refused permanent allowances by the State. It has always been in other wars a disgrace to the nation that many soldiers permanently disabled while upholding the honour of the country have, for want of adequate provision by the State, found their way into a workhouse. Mr. Bonar Law, at the Guildhall, bore eloquent testimony to the invaluable services rendered by our sailors and soldiers, whom he properly called "the children of the State." He pledged himself to move Parliament to make provision for the protection and maintenance of all who might be disabled in the present war. We hope many members of Parliament will make it their first business in the ensuing Session to remind Mr. Bonar Law of his pledge that the wrongs done to our warriors who are suffering from disablement other than actual wounds or loss of limbs shall speedily be remedied and an adequate allowance be made to them out of State funds.

The need for some arrangement for the wholesale sterilisation of cups, feeders, spoons, etc., used by hospital patients has long been recognised. Hitherto the tedious process of boiling them, a few at a time, in a saucepan, has seemed the only way to deal with the problem, and the news that a simple arrangement has been invented by Miss Chadburn whereby the kitchen sink can, by turning a tap, be converted into a steam-heated steriliser will be received with joy by hospital nursing staffs. These sink-sterilisers are to be seen in the ward kitchens of the new South London Hospital for Women, which is now nearing completion, and they look so delightfully simple that it is difficult to imagine why they have followed so far behind other kinds of sterilising apparatus.

There is a true ring about the story of the soldier and the junior probationer who was to be allowed to go with the case to the theatre. The former, rather a tough customer, had, after some weeks in hospital, had his leg amputated, and was just regaining consciousness; he was in a state of extreme collapse, and the sister, seeing that he wanted to speak, bent down to catch what he was trying to say, when, with some difficulty, he gasped out, "How did nurse stand the operation?"

Miss E. Small, matron, Royal Devon and Exeter Hospital, Exeter, has sent us a report of the Linen League of her hospital, of which Mrs. Ley is president. The eleventh annual meeting was held on the 11th inst., when it was announced that the contributors had maintained their usual numbers, and, despite the war, compared very favourably with those of previous years. 1,448 articles had been received, consisting chiefly of sheets, pillow-cases, blankets, towels, dishcloths, teacloths, tablecloths, bath towels, pieces of twill, nightdresses, stockings, aprons, and dusters. It is the custom at Exeter to exhibit all the work and linen in the Board-room. During the war the subscriptions and donations in actual money for the purchase of articles of the above description amounted to £129. All the linen used in the hospital for the last eleven years has been supplied by the League, which represents a saving in money equal to £200 a year. Further, the Linen League starts 1916 with a balance of upwards of £23 in hand. In offering our congratulations to the women of Exeter, who have accomplished this useful work, we should be glad to have particulars of the work accomplished by other Linen Leagues for voluntary hospitals. Mrs. Secretan is the hon. secretary of the Exeter Linen League. It is interesting to note in this connection that the audited accounts for the year ended December 31, 1915, show that, although the total gross expenditure exceeded the income by £1,198, Miss Small is able to report that some legacies have been paid in since the beginning of 1916 which more than meet this deficiency.

